# Migration characteristics of the Corail hydroxyapatite-coated femoral stem—a retrospective clinical evaluation and migration measurement with EBRA

**DOI:** 10.1007/s00402-021-03926-9

**Published:** 2021-05-17

**Authors:** Dietmar Dammerer, Philipp Blum, David Putzer, Dietmar Krappinger, Christof Pabinger, Michael C. Liebensteiner, Martin Thaler

**Affiliations:** 1 Department of Orthopaedics and Traumatology, Medical University of Innsbruck, Anichstrasse 35, 6020 Innsbruck, Austria; 2 Department of Experimental Orthopaedics, Medical University of Innsbruck, Sonnenburgstr. 16, 6020 Innsbruck, Austria; 3 Medical University of Innsbruck, Christoph-Probst-Platz 52, 6020 Innsbruck, Austria

**Keywords:** Stem subsidence, Total hip arthroplasty, Cementless, Einzel-Bild-Röntgen-Analyse (EBRA)

## Abstract

**Purpose:**

Uncemented stem migration analysis by EBRA-FCA (Einzel-Bild-Roentgen Analyse, Femoral Component Analysis) has been seen to be a good predictive indicator for early implant failure. In this study, we investigated the migration behavior of a cementless press-fit stem after two years follow-up. Stem type and postoperative gap between collar and femur were evaluated as a risk factor.

**Methods:**

Applying a retrospective study design, we reviewed all consecutive patients who between 2013 and 2017 received a cementless press-fit Corail stem (DePuy Orthopaedics Inc., Warsaw, IN, USA) at our Department. We reviewed medical histories and performed radiological measurements using EBRA-FCA software.

**Results:**

A total of 109 stems in 105 patients (female: 60; male: 45) fulfilled our inclusion criteria. Mean age at surgery was 67.8 (range, 21.6–90.5) years. EBRA migration analysis showed a mean subsidence of 1.8 mm (range, 0.0–12.1) at final follow-up. At 18 months mean subsidence of collared stems was significantly lower than in the collarless group [1.3 mm (range, 0.0–7.6) vs. 3.2 mm (range, 0.5–10.7), *p* = 0.0104]. Collared stems resting on the femoral cut presented a tendency to less subsidence than did collared stems showing a postoperative gap between collar and femur (1.3 vs. 2.0 mm) without finding statistical significance (*p* > 0.05).

**Conclusions:**

Low subsidence and the migration pattern of the cementless press-fit stem may predict a good long-term result. Collared stems investigated in our study provide good stability and are able to prevent significant subsidence.

*Trial registration number and date of registration:* Number: 20181024-1875**;** Date: 2018-10-24

## Introduction

Cemented as well as cementless femoral components in total hip arthroplasty (THA) yielded excellent long-term survival rates over 95% after ten years [1]. Nevertheless, the most common cause of failure in THA is aseptic loosening [2]. Previously published studies reported distal migration of the stem, called subsidence, which has shown to be a good predictive factor for early aseptic loosening [3–6]. According to Krismer et al. distal migration of the stem of more than 1.5 mm (mm) detected with EBRA-FCA within the first two years is a well-established risk factor for early implant failure [7]. However, comparability is limited due to the inclusion of cemented and cementless stems by Krismer et al. [7]. Streit et al. rated a limit of 2.7 mm axial migration as critical for the cementless CLS stem (Zimmer Inc, Warsaw, IN, USA) within the first two years after surgery [6].

EBRA-FCA is a computer-assisted method for measuring the distal migration of femoral stems using standard anterior–posterior (ap) pelvic radiographs without requiring additional means at exposure (e.g. ball markers). It has proven accuracy and a sensitivity of more than 1 mm in detecting migration, as compared to RSA (roentgen stereophotogrammetric analysis) [8, 9].

The stem investigated in this study is the Corail® stem by DuPuy Synthes (DePuy Orthopaedics Inc., Warsaw, IN, USA). It is designed for the cementless press-fit application, offers various offset options and is available with or without collar [10]. According to the Australian Orthopaedic Association National Joint Replacement Registry, 5283 Corail stems were implanted in Australia in 2018, which makes it the most used cementless stem in primary THA [11].

In the present study, we investigated the clinical results and the migration behavior of the cementless Corail stem using EBRA-FCA with a follow-up of 24 months. Furthermore, we evaluated the possible influence of stem type and distance between collar and femur on stem subsidence.

## Material and methods

The study was approved by the local ethics committee (Medical University of Innsbruck, Austria, Europe). We applied a retrospective study design and reviewed all consecutive patients who received a Corail stem at our Department between 2013 and 2017. During this time, a total of 217 Corail stems were implanted as part of a primary THA. The type of Corail stem was chosen by the surgeon depending on the patient’s situation.

We investigated the medical histories for sociodemographic data, surgical approach, pre- and postoperative range of motion, body mass index, cut-to-suture time and the preoperative diagnosis for THA indication. Furthermore, the estimated blood loss was calculated using the formula of Mercuriali [12].

**Fig. 1:**
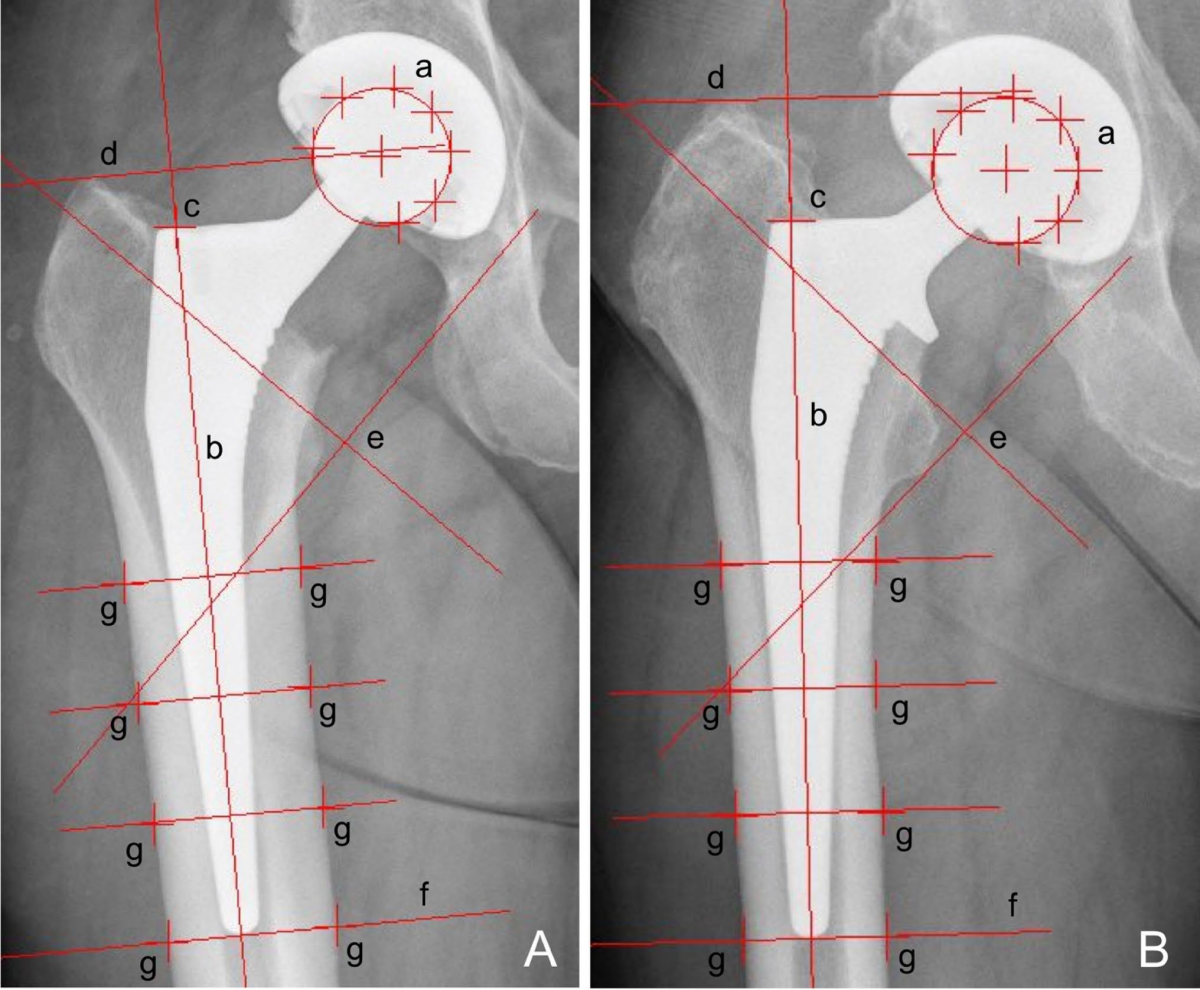
Anterior to posterior x rays showing a collarless (**A**) and a collared (**B**) Corail stem with EBRA-FCA references **a** head points **b** stem axis **c** stem shoulder **d** major trochanter line **e** minor trochanter lines **f** tip-of-stem line **g** points at femoral bone contour

Axial stem migration and prosthetic stability of the stem were assessed retrospectively with EBRA-FCA (German: Einzel-Bild-Röntgen-Analyse, Femoral Component Analysis) from plain x rays [7, 8]. A total of 19 reference points are defined on the femoral head (*n* = 7), the stem (*n* = 2), the femoral cortex (*n* = 8), and one each at the major and minor trochanter [8]. The EBRA-FCA software excludes radiographs with a comparability algorithm, which identifies significant positioning artifacts by comparing specific bone and prosthetic landmarks. Figure 1 shows the x-rays of a collared and a collarless Corail stem including EBRA-FCA references.

We followed patients with radiographs before discharge, six weeks after surgery, 12 months postoperatively and at the subsequent annual check-ups. Additional radiographs were performed if the patient voiced complaints after THA. All radiographs were taken with the same technique and following the EBRA protocol: anterior–posterior (AP) radiographs; patient standing in upright position and full weight-bearing. For EBRA-FCA analysis, a minimum of four radiographs per patient and a minimum of eight months radiological follow-up were required. Stem migration analysis was performed with EBRA-FCA by one independent investigator, who was not involved in the surgeries or postoperative treatment of the patients. The head sizes used for EBRA-FCA calibration were taken from the operation notes.

In addition, the influence that stem type (collared vs. collarless) and distance between collar and femur (0 vs. > 0 mm) exerted on subsidence was investigated.

## Statistics

Mean, median, range, and standard deviation were calculated for the various measurement parameters. For the analysis, Access and Excel (Microsoft Office Professional Plus 2010, Redmond, WA, USA) as well as Graph Pad Prism (Version 8.0, GraphPad Software, Inc., La Jolla, CA, USA) were used. Subsidence was classified as greater or less than 1.5 or 2.7 mm at 2-year follow-up [6, 7]. All data were tested for normality using the Kolmogorov–Smirnov test. For comparison of the EBRA measurements at different time steps the Kruskal Wallis test was used. The EBRA measurements were compared by stem type using the Mann Whitney U-Test. When comparing the range of motion pre- and postoperatively the Mann Whitney U test was used. A p value of 0.05 was considered statistically significant.

## Results

A total of 109 stems in 105 patients (female: 60; male: 45) fulfilled our inclusion criteria. Of these 32 were collared standard stems, 40 collared high-offset stems, 7 collared coxa vara stems, 11 collarless standard stems and 13 collarless high-offset stems. For another six collared stems the offset version could not be assessed. In four patients a Corail stem was implanted bilaterally. Mean follow-up was 25.1 (range, 8–57) months. The preoperative diagnosis was osteoarthritis in 104 (95.4%) hips, avascular necrosis of the femoral head in four (3.7%) hips and hip dysplasia with secondary osteoarthritis in one (0.9%) hip. The investigated stem was combined with a cementless press-fit Pinnacle cup (DePuy Orthopaedics Inc., Warsaw, IN, USA). The most used stem and head sizes were 12 (22.9%) and 32 mm (89.9%), respectively. More details on patients’ demographics and surgical procedure are shown in Tables 1 and 2.

**Table 1:** Patient demographics for the study group. Range is given in brackets

Number of patients	Female	60
	Male	45
	Total	105
Mean age (years)		67.8 (21.6–90.5)
BMI (kg/m2)		26.8 (17.4–50.8)
Cut-to-suture time (min)		80 (36–200)
Surgical approach	Direct anterior approach	107
	Anterolateral approach	2
Surgical position	Supine	109
Preoperative diagnosis	Osteoarthritis	104
	Avascular necrosis of the femoral head	4
	Hip dysplasia	1
Total blood loss (l)		1.2 (0.1–5.0)

**Table 2:** Details of implanted components. Percentages are given in brackets

Stem product	Corail	109 [100.0]
Stem type	Collared	85 [78.0]
	Collarless	24 [22.0]
Stem offset	Standard	43 [39.5]
	High-offset	53 [48.6]
	Coxa vara	7 [6.4]
	n.a	6 [5.5]
Cup product	Pinnacle	109 [100.0]

*n.a.* not available

EBRA-FCA analysis at 24 months follow-up was calculated for 67 of the 109 stems with an EBRA-FCA-given comparability limit of 3.0 mm (95% confidence interval). A total of 469 × rays were analyzed, 29 (6.2%) radiographs rejected by the EBRA-FCA software. On average, 4.3 (range, 4–7) x rays per implant were analyzed. None of our patients had to be excluded from EBRA-FCA migration analysis. A complete set of radiographs at every single time step (e.g. six months, 12 months, etc.) was not available for each stem in our study. Therefore, total subsidence could not be calculated for all cases. This gives a different number of cases in the corresponding migration behavior analysis over time.

**Fig. 2:**
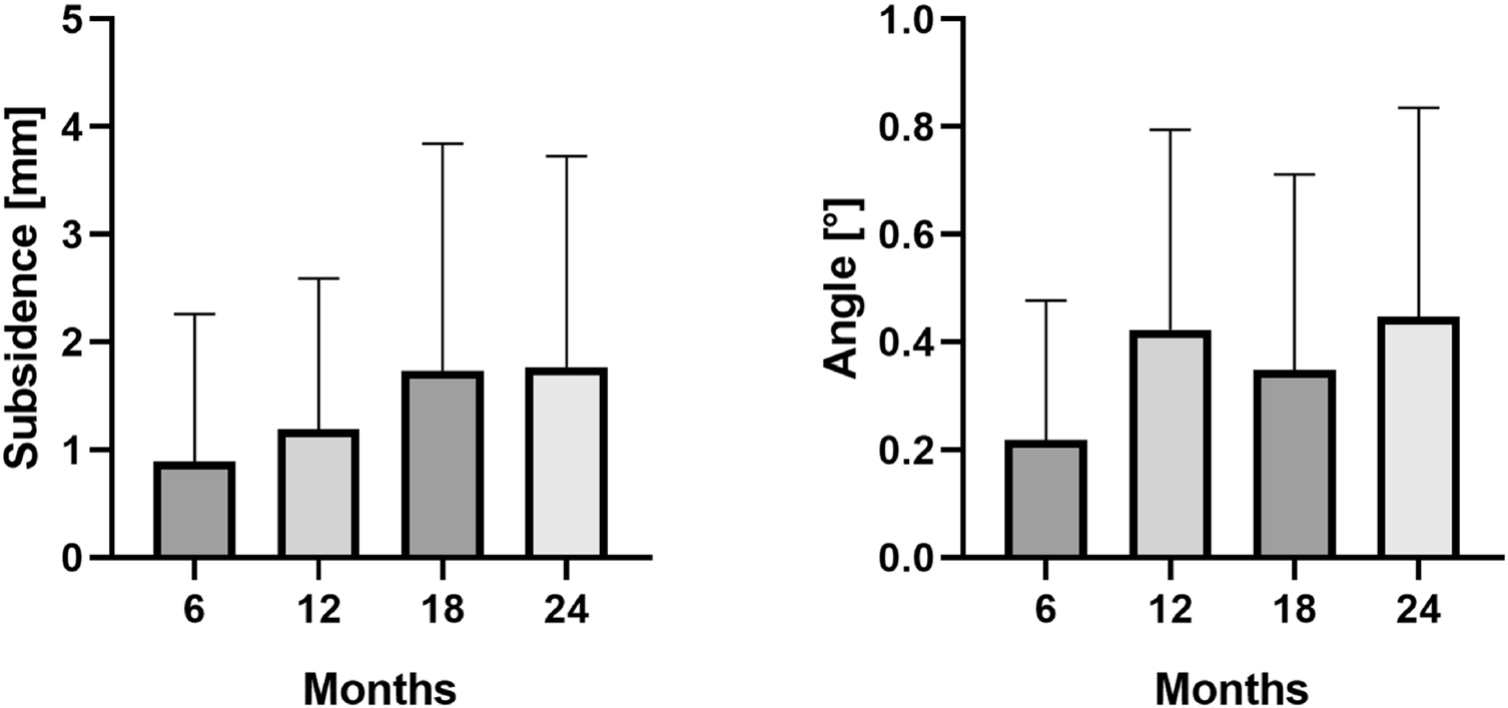
Mean and standard deviation (bars) of the measured subsidence and the angle between stem and anatomical femoral axis over the follow-up period of 24 months

The EBRA-FCA analysis showed a mean migration of 0.9 mm (median 0.4; range 0.0–8.0) at six months, 1.2 mm (median 0.7; range 0.0–7.5) at 12 months, 1.7 mm (median 1.1; range 0.0–10.7) at 18 months, 1.8 mm (median 1.3; range 0.0–12.1) at 24 months after surgery. Thus, the main axial subsidence occurred particularly in the first 18 months postoperatively (Table 3 and Fig. 2). The calculated mean monthly axial implant migration was 0.15 mm within the first six months, 0.05 mm between six and 12 months, 0.09 mm between 12 and 18 months and less than 0.01 mm between 18 and 24 months after surgery. A statistically significant difference was found between six and 18 months (p = 0.0065) and between six and 24 months (*P* < 0.0001). No statistically significant difference was found for any other subsidence measurements (*p* > 0.05).

**Table 3:** Mean total subsidence in millimeters (mm) over time. Range is given in brackets

	6 months (n = 91)	12 months (n = 57)	18 months (n = 49)	24 months (n = 67)
Subsidence of the Corail stem in mm (range)	0.9 (0.0–8.0)	1.2 (0.0–7.5)	1.7 (0.0–10.7)	1.8 (0.0–12.1)

Percentages of the migrated stems are given in Table 4.

**Table 4:** Total subsidence in millimeters (mm) over time. Percentages are given in square brackets

Total subsidence (mm)	6 months (*n* = 91)	12 months (*n* = 57)	18 months (*n* = 49)	24 months (*n* = 67)
≤ 1.5	74 [81.3]	40 [70.2]	32 [65.3]	41 [61.2]
> 1.5	10 [11.0]	9 [15.8]	7 [14.3]	16 [23.9]
> 2.7	7 [7.7]	8 [14.0]	10 [20.4]	10 [14.9]

In addition, the angle between stem and femur axis was 0.2° (median 0.1°; range 0.0°–1.1°) after six months, 0.4° (median 0.4°; range 0.0°–1.5°) after 12 months, 0.3° (median 0.2°; range 0.0°–1.2°) after 18 months and 0.4° (median 0.3°; range 0.0°–1.9°) after 24 months (Fig. 2). A statistically significant difference was found between six and 12 months (*p* = 0.0013) and between six and 24 months (*p* < 0.0001). No statistically significant difference could be found for any other angle measurements (*p* > 0.05).

**Fig. 3:**
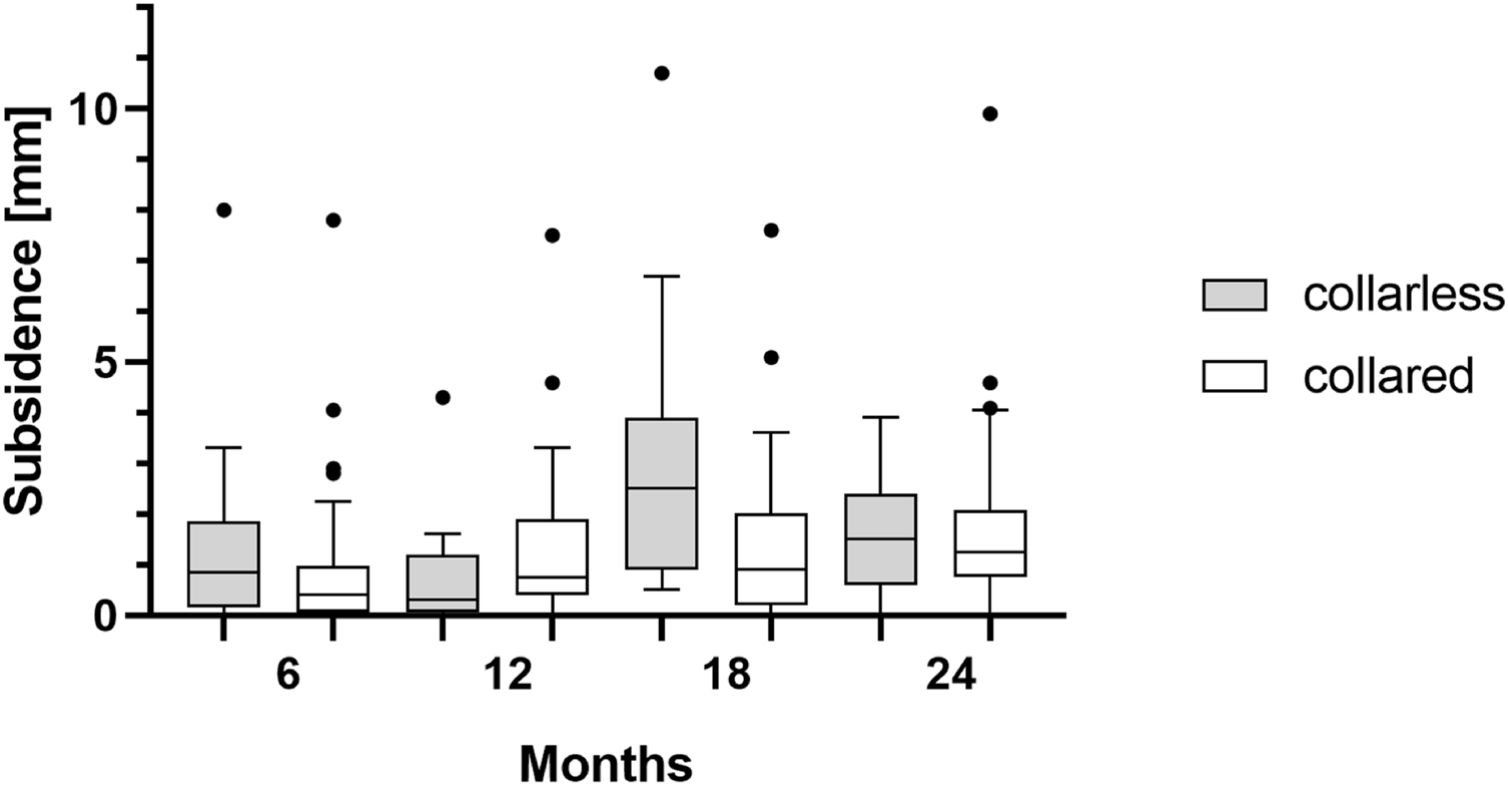
Boxplots of the measured subsidence are shown for collared and collarless implants. Whiskers and outliers (dots) were determined according to the Tuckey method

Subgroup analysis of collared and collarless implants (Fig. 3) showed a mean subsidence of 0.7 mm (median 0.4; range 0.0–7.8) vs. 1.4 mm (median 0.9; range 0.0–8.0) after six months, 1.3 mm (median 0.8; range 0.0–7.5) vs. 0.9 mm (median 0.3; range 0.0–4.3) after 12 months, 1.3 mm (median 0.9; range 0.0–7.6) vs. 3.2 mm (median 2.5; range 0.5–10.7) after 18 months and 1.6 mm (median 1.3; range 0.0–9.9) vs. 2.2 mm (median 1.5; range 0.0–12.1) after 24 months.

**Fig. 4:**
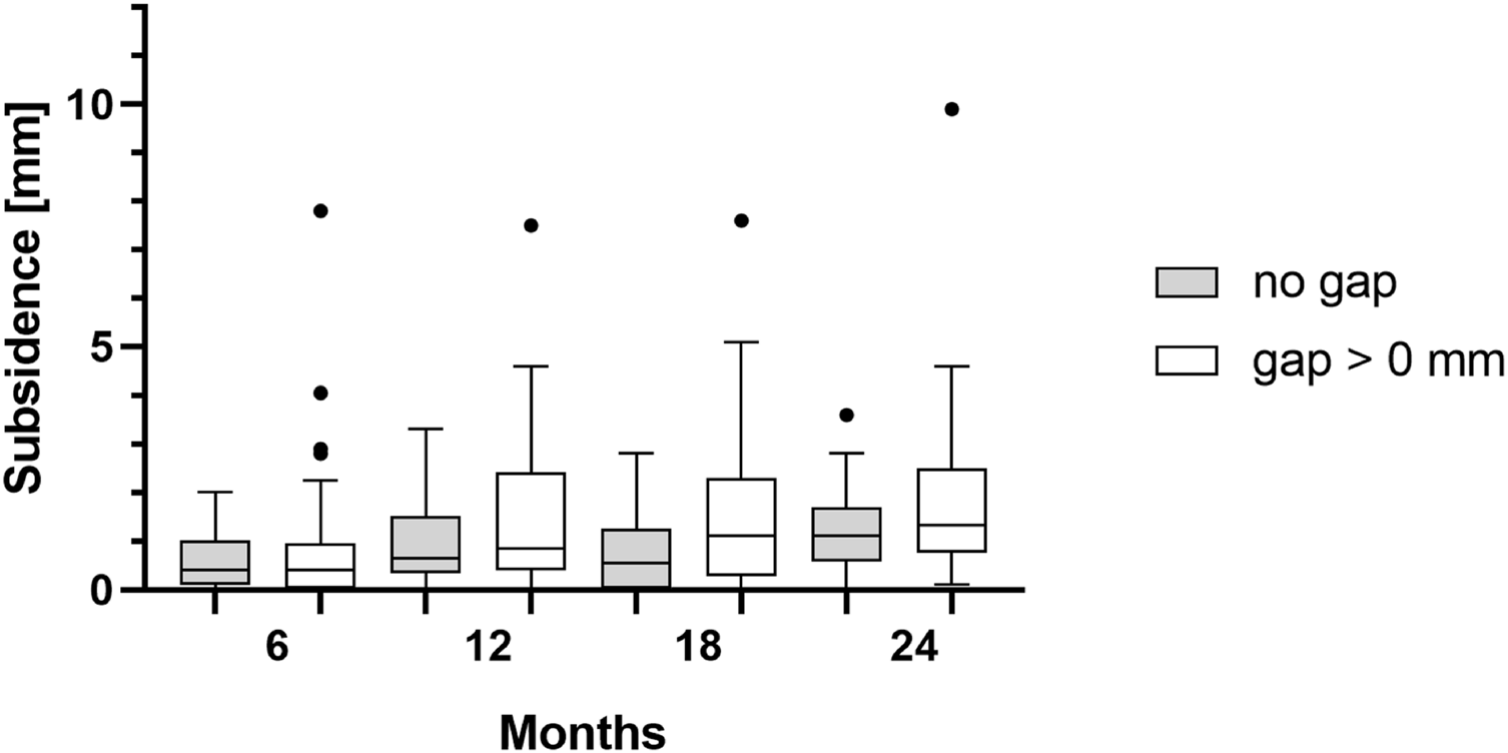
Boxplots of the measured subsidence are shown for collared implants with and without a gap between collar and osteotomy level. Whiskers and outliers (dots) were determined according to the Tuckey method

In addition, 40 of 85 (47.1%) collared implants had immediate postoperative contact between the collar and the femoral cut. Collared implants presenting a postoperative gap between collar and femur revealed greater mean subsidence than did collar implants without gap after 24 months (2.0 mm vs. 1.3 mm; median 1.3 mm vs. 1.1 mm). However, no statistically significant differences could be found (*p* > 0.05). Further migration values at different time steps are shown in Fig. 4.

Pre- and postoperative comparison of the range of motion showed a mean improvement in flexion of 14° (range, − 40°–40°, *p* < 0.0001), internal rotation 11° (range, − 40°–40°, *p* < 0.0001), external rotation 7° (range, -15°–45°, *p* < 0.0001) and abduction 7° (range, -30°–30°, *p* < 0.0001). No statistically significant improvement was found for hip adduction when comparing pre- and postoperative range of motion (*p* = 0.1391). While preoperatively a flexion ≥ 90° was possible in only 74.3% of the hips, this increased to 94.5% postoperatively.

## Discussion

As far as we know, this is the first study to characterize the migration behavior of the Corail stem using EBRA-FCA software. The most important finding of our study is a mean subsidence of 1.8 mm after a follow-up of 24 months. While the subsidence rate of collared stems was lower (collared 1.6 mm vs. collarless 2.2 mm, *p* > 0.05) at 24 months, a statistically significant difference was only found between groups at 18 months (*p* = 0.0104). Furthermore, collared stems without a postoperative gap between collar and femur showed a tendency to less subsidence than collared stems with a gap did.

The use of cementless stems requires a high level of press fit to achieve primary implant stability [13]. Otherwise, subsidence can lead to aseptic loosening of the implant [7]. With a specificity of 100% and a sensitivity of 78% as compared with roentgen stereophotogrammetric analysis (RSA) for detection of migration of more than 1 mm, EBRA-FCA is suitable for identifying and measuring the subsidence of femoral components in THA [8]. While RSA is considered to be the gold standard for migration measurement, EBRA offers the advantage of being a non-invasive method that can be used in our retrospective study design.

Several studies have already evaluated the subsidence of cementless stems using a variety of measurement techniques [14–19]. When investigating 30 collarless, standard offset Corail stems with RSA, Campbell et al. presented a mean migration of 0.58 mm after two years with the main subsidence occurring within the first six months [15]. In contrast, the mean subsidence measured by Ries et al. in 231 collared and collarless Corail stems amounted to 2.9 mm after a mean follow-up of seven months [18]. Subsidence was measured as the distance between stem shoulder and major trochanter [18]. EBRA-FCA analysis by Stihsen et al. showed a mean subsidence of 1.38 mm for 105 cementless Vision 2000 stems (DePuy, Warsaw, IN, USA) [20]. Compared to these results, we can present a mean subsidence of 1.8 mm after two years. In our study, the main subsidence occurred in the first six months, showing an axial migration of 0.15 mm/month, and we can thus confirm the observation made by Campbell et al. [15]. Selvaratnam et al. and Al-Najim et al., who monitored the Corail stem at shorter intervals, reported that the main subsidence occurred in the first six weeks after surgery [21, 22]. Selvaratnam et al. hypothesized that early migration of cementless Corail stems is a form of impaction rather than a real subsidence leading to implant instability [21]. The phenomenon of early subsidence with later stabilization is not unknown and was already described in 1999 by Krismer et al. [7]. It is assumed that secondary stabilization can lead to long-lasting survival of the implant [7].

The Corail stem is available with or without a collar. As mentioned by the company, collared stems are available to prevent subsidence and provide additional rotational stability in patients with osteopenic bone [10]. In 1997, Meding et al. investigated collared and collarless femoral stems in uncemented primary THA without finding significant differences in adequacy of fixation or clinical scores [14]. However, Demey et al. performed a comparative bilateral cadaver study showing significantly greater immediate stability of collared stems, in that they withstood greater vertical and horizontal forces before subsidence occurred [17]. Thanks to this benefit, Demey et al. routinely use collared stems in uncemented primary THA [17]. Ries et al. confirmed the findings of Demey et al. by showing a significantly greater subsidence of collarless than of collared Corail stems (3.1 mm vs. 1.6 mm) after a mean follow-up of seven months [18]. In our study, the group of collarless Corail stems also showed greater mean subsidence than did the collared stems after two years of follow-up (2.2 vs. 1.6 mm, respectively), whereby statistical significance was found only for measurements at 18 months.

In addition to the used stem type, biomechanical tests show that the immediate postoperative distance between collar and femur seems to influence the primary stability of the implant [17]. Al-Najim et al. suggested that further stem subsidence is prevented once the collar is in contact with the medial femoral cut [22]. While in the Meding et al. study group 39% of the stem collars rested on the femur [14], this figure was 47.1% in our study. We found a lower mean subsidence of collared stems with immediate postoperative contact to the femoral cut than of stems without after two years follow-up (1.3 vs. 2.0 mm). Although no statistically significant differences were found, the detected tendency at all measurement steps leads us to hypothesize that a collar resting on the femoral cut might prevent increased subsidence.

This study has several limitations, including the retrospective methodology. As a result, some of the treated patients had to be excluded from the cohort, possibly making the study more prone to selection bias. Furthermore, there were a varying number of radiographs and duration of follow-up for each hip. This may have influenced the migration results due to the smoothing function within the software and made it difficult to follow the exact outcome of each individual implant. The unequal distribution of collared and collarless cases has to be mentioned as a possible bias, which may have influenced the final result. In addition, we did not compare our results with another patient cohort using a different stem design. However, migration analysis results are well published, and therefore our results can be compared with previously published results, which, however, did not compare their results with those of another patient cohort. Furthermore, some patient characteristics (e.g. smoking, osteoporosis), which might have influenced the clinical outcome of the implant, could not be assessed.

In conclusion, EBRA-FCA analysis of the Corail stem showed low mean subsidence, reduction of migration speed and low tilting of the stem with good clinical function at 24 months. At 18 months mean subsidence of collared stems was significantly lower than for the collarless group. Collared stems resting on femoral cut presented a tendency to less subsidence than did collared stems showing a postoperative gap between collar and femur. Further investigations of a larger cohort are necessary to identify possible clinical differences in outcome between the two groups over the long term.

## Data Availability

Data will be sent if necessary.
